# (*E*)-2-Acetyl­pyrazine 4-nitro­phenyl­hydrazone

**DOI:** 10.1107/S1600536808017479

**Published:** 2008-06-13

**Authors:** Shang Shan, Yu-Liang Tian, Shan-Heng Wang, Wen-Long Wang, Ying-Li Xu

**Affiliations:** a College of Chemical Engineering and Materials Science, Zhejiang University of Technology, People’s Republic of China

## Abstract

In the title compound, C_12_H_11_N_5_O_2_, the mol­ecule adopts an *E* configuration, with the benzene and pyrazine rings located on opposite sides of the N=C double bond. The face-to-face separations of 3.413 (14) and 3.430 (8) Å, respectively between parallel benzene rings and between pyrazine rings indicate the existence of π–π stacking between adjacent mol­ecules. The crystal structure also contains N—H⋯N and C—H⋯O hydrogen bonding.

## Related literature

For general background, see: Okabe *et al.* (1993 ▶); Hu *et al.* (2001 ▶); Chen *et al.* (2007 ▶). For a related structure, see: Shan *et al.* (2008 ▶).
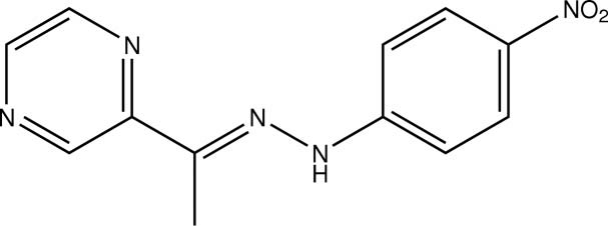


## Experimental

### 

#### Crystal data

C_12_H_11_N_5_O_2_
*M* *_r_* = 257.26
Monoclinic, 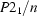

*a* = 8.0101 (6) Å
*b* = 12.5154 (11) Å
*c* = 12.1506 (12) Å
β = 98.564 (2)°
*V* = 1204.51 (18) Å^3^
*Z* = 4
Mo *K*α radiation
μ = 0.10 mm^−1^
*T* = 295 (2) K
0.40 × 0.38 × 0.26 mm

#### Data collection

Rigaku R-AXIS RAPID IP diffractometer
Absorption correction: none
11633 measured reflections
2747 independent reflections
1446 reflections with *I* > 2σ(*I*)
*R*_int_ = 0.033

#### Refinement

*R*[*F* ^2^ > 2σ(*F* ^2^)] = 0.042
*wR*(*F* ^2^) = 0.149
*S* = 1.08
2747 reflections
174 parameters
H-atom parameters constrained
Δρ_max_ = 0.20 e Å^−3^
Δρ_min_ = −0.19 e Å^−3^

### 

Data collection: *PROCESS-AUTO* (Rigaku, 1998 ▶); cell refinement: *PROCESS-AUTO*; data reduction: *CrystalStructure* (Rigaku, 2002 ▶); program(s) used to solve structure: *SIR92* (Altomare *et al.*, 1993 ▶); program(s) used to refine structure: *SHELXL97* (Sheldrick, 2008 ▶); molecular graphics: *ORTEP-3 for Windows* (Farrugia, 1997 ▶); software used to prepare material for publication: *WinGX* (Farrugia, 1999 ▶).

## Supplementary Material

Crystal structure: contains datablocks I, global. DOI: 10.1107/S1600536808017479/sg2248sup1.cif

Structure factors: contains datablocks I. DOI: 10.1107/S1600536808017479/sg2248Isup2.hkl

Additional supplementary materials:  crystallographic information; 3D view; checkCIF report

## Figures and Tables

**Table 1:** Hydrogen-bond geometry (Å, °)

*D*—H⋯*A*	*D*—H	H⋯*A*	*D*⋯*A*	*D*—H⋯*A*
N2—H2*N*⋯N5^i^	0.91	2.30	3.185 (2)	164
C11—H11⋯O1^ii^	0.93	2.60	3.300 (3)	133

Symmetry codes: (i) 
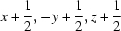
; (ii) 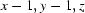.

**Fig. 1.**
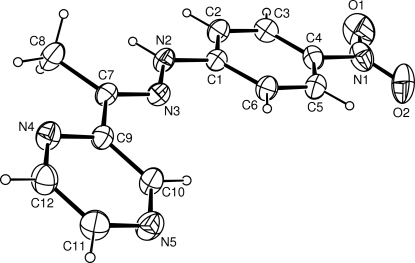
The molecular structure of (I) with 30% probability displacement ellipsoids (arbitrary spheres for H atoms), dashed line indicates hydrogen bonding.

**Fig. 2.**
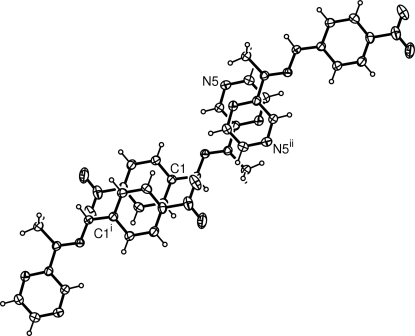
A diagram showing π-π stacking [symmetry codes: (i) 2 - x,1 - y,1 - z; (ii) 1 - x,-y,1 - z].

## supplementary crystallographic information

### Comment

Hydrazone and its derivatives have attracted  much attention because of their potential application in biology (Okabe *et al.*, 1993; Hu *et al.*, 2001). As part of an ongoing investigation into anti-cancer compounds (Chen *et al.*, 2007), the title compound has recently been prepared in our laboratory and its crystal structure is presented here.

The molecular structure of the title compound is shown in Fig. 1. The molecule adopts an  E-configuration, with the benzene and pyrazine rings located on the opposite positions of the N3=C7 double bond, similar to that found in a related structure, (E)-2-Furyl methyl ketone 2,4-dinitrophenylhydrazone (Shan *et al.*, 2008). The pyrazine plane is twisted with respect to the benzene ring by a smaller dihedral angle of 14.25 (10)°.

The partially overlapped arrangement is observed between parallel benzene rings and between parallel pyrazine rings (Fig. 2), face-to-face separations of 3.413 (14) [for benzene rings] and 3.430 (8) Å [for pyrazine rings] are significantly shorter than van der Waals thickness of the aromatic ring (3.70 Å), and indicate the existence of π-π stacking between the adjacent molecules. Intermolecular  N—H···N and weak C—H···O hydrogen bondings are present in the crystal structure (Table 1).

### Experimental

4-Nitrophenylhydrazine (0.31 g, 2 mmol) was dissolved in ethanol (10 ml), then H_2_SO_4_ solution (98%, 0.5 ml) was added slowly to the ethanol solution with stirring. The solution was heated at about 333 K for several minutes until the solution cleared. An ethanol solution (5 ml) of acetylpyrazine (0.24 g, 2 mmol) was dropped slowly into the above solution with continuous stirring, and the mixture solution was kept at about 333 K for 0.5 h. When the solution had cooled to room temperature, yellow microcrystals appeared. They were separated and washed with cold water three times to get the product 0.40 g. Single crystals of the title compound were obtained by recrystallization from an absolute ethanol solution.

### Refinement

Methyl H atoms were placed in calculated positions with C—H = 0.96 Å and torsion angle was refined to fit the electron density, *U*_iso_(H) = 1.5*U*_eq_(C). Imino H atom was located in a difference Fourier map and refined as riding in its as-found relative position, *U*_iso_(H) = 1.2*U*_eq_(N). Aromatic H atoms were placed in calculated positions with C—H = 0.93 and refined in riding mode with *U*_iso_(H) = 1.2*U*_eq_(C).

### Crystal data

**Table:**

C_12_H_11_N_5_O_2_	*F*_000_ = 536
*M**_r_* = 257.26	*D*_x_ = 1.419 Mg m^−^^3^
Monoclinic, *P*2_1_/*n*	Melting point: 498 K
Hall symbol: -P 2yn	Mo *K*α radiation λ = 0.71069 Å
*a* = 8.0101 (6) Å	Cell parameters from 4236 reflections
*b* = 12.5154 (11) Å	θ = 3.2–25.0º
*c* = 12.1506 (12) Å	µ = 0.10 mm^−^^1^
β = 98.564 (2)º	*T* = 295 (2) K
*V* = 1204.51 (18) Å^3^	Prism, yellow
*Z* = 4	0.40 × 0.38 × 0.26 mm

### Data collection

**Table:**

Rigaku R-AXIS RAPID IP diffractometer	2747 independent reflections
Radiation source: fine-focus sealed tube	1446 reflections with *I* > 2σ(*I*)
Monochromator: graphite	*R*_int_ = 0.033
Detector resolution: 10.00 pixels mm^-1^	θ_max_ = 27.4º
*T* = 295(2) K	θ_min_ = 3.0º
ω scans	*h* = −10→10
Absorption correction: none	*k* = −16→16
11633 measured reflections	*l* = −15→15

### Refinement

**Table:**

Refinement on *F*^2^	Hydrogen site location: inferred from neighbouring sites
Least-squares matrix: full	H-atom parameters constrained
*R*[*F*^2^ > 2σ(*F*^2^)] = 0.042	*w* = 1/[σ^2^(*F*_o_^2^) + (0.0615*P*)^2^ + 0.2787*P*] where *P* = (*F*_o_^2^ + 2*F*_c_^2^)/3
*wR*(*F*^2^) = 0.149	(Δ/σ)_max_ = 0.001
*S* = 1.09	Δρ_max_ = 0.20 e Å^−^^3^
2747 reflections	Δρ_min_ = −0.19 e Å^−^^3^
174 parameters	Extinction correction: SHELXL97 (Sheldrick, 2008), Fc^*^=kFc[1+0.001xFc^2^λ^3^/sin(2θ)]^-1/4^
Primary atom site location: structure-invariant direct methods	Extinction coefficient: 0.024 (3)
Secondary atom site location: difference Fourier map	

### Special details

**Table:**

Geometry. All e.s.d.'s (except the e.s.d. in the dihedral angle between two l.s. planes) are estimated using the full covariance matrix. The cell e.s.d.'s are taken into account individually in the estimation of e.s.d.'s in distances, angles and torsion angles; correlations between e.s.d.'s in cell parameters are only used when they are defined by crystal symmetry. An approximate (isotropic) treatment of cell e.s.d.'s is used for estimating e.s.d.'s involving l.s. planes.
Refinement. Refinement of *F*^2^ against ALL reflections. The weighted *R*-factor *wR* and goodness of fit *S* are based on *F*^2^, conventional *R*-factors *R* are based on *F*, with *F* set to zero for negative *F*^2^. The threshold expression of *F*^2^ > σ(*F*^2^) is used only for calculating *R*-factors(gt) *etc*. and is not relevant to the choice of reflections for refinement. *R*-factors based on *F*^2^ are statistically about twice as large as those based on *F*, and *R*- factors based on ALL data will be even larger.

### Fractional atomic coordinates and isotropic or equivalent isotropic displacement parameters (Å^2^)

**Table:**

	*x*	*y*	*z*	*U*_iso_*/*U*_eq_	
N1	0.9003 (3)	0.68785 (16)	0.3418 (2)	0.0684 (6)	
N2	0.6332 (2)	0.36085 (12)	0.57394 (13)	0.0448 (4)	
H2N	0.6470	0.3652	0.6496	0.054*	
N3	0.5365 (2)	0.28334 (12)	0.51838 (13)	0.0425 (4)	
N4	0.3197 (2)	0.04376 (14)	0.55830 (14)	0.0518 (5)	
N5	0.2175 (2)	0.07812 (15)	0.33069 (14)	0.0571 (5)	
O1	0.9806 (3)	0.75846 (16)	0.3955 (2)	0.1041 (8)	
O2	0.8729 (3)	0.68826 (16)	0.2397 (2)	0.1012 (8)	
C1	0.6976 (2)	0.43994 (15)	0.51344 (16)	0.0412 (5)	
C2	0.7855 (3)	0.52394 (16)	0.57162 (17)	0.0490 (5)	
H2	0.7989	0.5251	0.6490	0.059*	
C3	0.8519 (3)	0.60439 (16)	0.51558 (18)	0.0521 (5)	
H3	0.9109	0.6601	0.5544	0.062*	
C4	0.8306 (3)	0.60198 (15)	0.40117 (18)	0.0488 (5)	
C5	0.7461 (3)	0.51920 (17)	0.34168 (17)	0.0513 (5)	
H5	0.7339	0.5187	0.2644	0.062*	
C6	0.6803 (3)	0.43744 (17)	0.39758 (16)	0.0482 (5)	
H6	0.6246	0.3809	0.3583	0.058*	
C7	0.4778 (2)	0.20837 (14)	0.57459 (15)	0.0408 (5)	
C8	0.5120 (3)	0.19727 (18)	0.69835 (17)	0.0593 (6)	
H8A	0.6317	0.1939	0.7222	0.089*	
H8B	0.4602	0.1331	0.7202	0.089*	
H8C	0.4663	0.2578	0.7322	0.089*	
C9	0.3700 (2)	0.13023 (15)	0.50718 (15)	0.0408 (5)	
C10	0.3179 (3)	0.14575 (16)	0.39353 (16)	0.0496 (5)	
H10	0.3553	0.2063	0.3602	0.059*	
C11	0.1699 (3)	−0.00860 (18)	0.38283 (19)	0.0569 (6)	
H11	0.1002	−0.0587	0.3424	0.068*	
C12	0.2214 (3)	−0.02519 (17)	0.49398 (19)	0.0554 (6)	
H12	0.1865	−0.0871	0.5263	0.067*	

### Atomic displacement parameters (Å^2^)

**Table:**

	*U*^11^	*U*^22^	*U*^33^	*U*^12^	*U*^13^	*U*^23^
N1	0.0742 (15)	0.0486 (11)	0.0895 (17)	0.0037 (10)	0.0352 (12)	0.0135 (11)
N2	0.0566 (11)	0.0430 (9)	0.0341 (8)	−0.0036 (8)	0.0039 (7)	−0.0005 (7)
N3	0.0478 (10)	0.0391 (8)	0.0397 (9)	0.0001 (7)	0.0036 (7)	−0.0009 (7)
N4	0.0623 (12)	0.0469 (10)	0.0465 (10)	−0.0033 (8)	0.0094 (8)	0.0044 (8)
N5	0.0678 (13)	0.0578 (11)	0.0435 (10)	−0.0075 (9)	0.0012 (9)	−0.0042 (9)
O1	0.1140 (18)	0.0645 (12)	0.137 (2)	−0.0350 (12)	0.0270 (15)	0.0119 (13)
O2	0.146 (2)	0.0841 (14)	0.0860 (15)	−0.0086 (13)	0.0569 (14)	0.0232 (12)
C1	0.0454 (11)	0.0388 (10)	0.0393 (10)	0.0033 (8)	0.0064 (8)	−0.0002 (8)
C2	0.0588 (13)	0.0456 (11)	0.0419 (11)	−0.0015 (10)	0.0050 (9)	−0.0038 (9)
C3	0.0542 (13)	0.0412 (11)	0.0609 (14)	−0.0029 (9)	0.0087 (10)	−0.0062 (10)
C4	0.0517 (12)	0.0405 (10)	0.0577 (13)	0.0055 (9)	0.0198 (10)	0.0075 (10)
C5	0.0610 (14)	0.0527 (12)	0.0414 (11)	0.0029 (10)	0.0117 (10)	0.0022 (10)
C6	0.0569 (13)	0.0471 (11)	0.0411 (11)	−0.0013 (10)	0.0088 (9)	−0.0023 (9)
C7	0.0469 (12)	0.0381 (10)	0.0370 (10)	0.0050 (8)	0.0046 (8)	0.0027 (8)
C8	0.0780 (17)	0.0573 (13)	0.0396 (12)	−0.0086 (12)	−0.0009 (11)	0.0065 (10)
C9	0.0459 (11)	0.0391 (10)	0.0379 (10)	0.0036 (8)	0.0079 (8)	0.0035 (8)
C10	0.0605 (14)	0.0483 (11)	0.0394 (11)	−0.0045 (10)	0.0060 (10)	0.0024 (9)
C11	0.0619 (15)	0.0520 (13)	0.0561 (13)	−0.0091 (11)	0.0064 (11)	−0.0063 (11)
C12	0.0634 (14)	0.0473 (12)	0.0568 (13)	−0.0107 (10)	0.0127 (11)	0.0019 (11)

### Geometric parameters (Å, °)

**Table:**

N1—O1	1.223 (3)	C3—H3	0.9300
N1—O2	1.226 (3)	C4—C5	1.381 (3)
N1—C4	1.452 (3)	C5—C6	1.376 (3)
N2—N3	1.357 (2)	C5—H5	0.9300
N2—C1	1.378 (2)	C6—H6	0.9300
N2—H2N	0.9106	C7—C9	1.470 (3)
N3—C7	1.290 (2)	C7—C8	1.494 (3)
N4—C12	1.339 (3)	C8—H8A	0.9600
N4—C9	1.339 (2)	C8—H8B	0.9600
N5—C10	1.328 (3)	C8—H8C	0.9600
N5—C11	1.340 (3)	C9—C10	1.395 (3)
C1—C6	1.394 (3)	C10—H10	0.9300
C1—C2	1.397 (3)	C11—C12	1.368 (3)
C2—C3	1.367 (3)	C11—H11	0.9300
C2—H2	0.9300	C12—H12	0.9300
C3—C4	1.375 (3)		
			
O1—N1—O2	122.5 (2)	C5—C6—C1	119.6 (2)
O1—N1—C4	118.7 (2)	C5—C6—H6	120.2
O2—N1—C4	118.8 (2)	C1—C6—H6	120.2
N3—N2—C1	118.65 (15)	N3—C7—C9	114.78 (16)
N3—N2—H2N	121.3	N3—C7—C8	125.00 (18)
C1—N2—H2N	119.8	C9—C7—C8	120.23 (17)
C7—N3—N2	118.88 (16)	C7—C8—H8A	109.5
C12—N4—C9	116.19 (18)	C7—C8—H8B	109.5
C10—N5—C11	115.78 (18)	H8A—C8—H8B	109.5
N2—C1—C6	122.25 (18)	C7—C8—H8C	109.5
N2—C1—C2	118.10 (17)	H8A—C8—H8C	109.5
C6—C1—C2	119.64 (19)	H8B—C8—H8C	109.5
C3—C2—C1	120.42 (19)	N4—C9—C10	120.35 (18)
C3—C2—H2	119.8	N4—C9—C7	118.11 (16)
C1—C2—H2	119.8	C10—C9—C7	121.53 (17)
C2—C3—C4	119.2 (2)	N5—C10—C9	123.13 (19)
C2—C3—H3	120.4	N5—C10—H10	118.4
C4—C3—H3	120.4	C9—C10—H10	118.4
C3—C4—C5	121.53 (19)	N5—C11—C12	121.6 (2)
C3—C4—N1	119.1 (2)	N5—C11—H11	119.2
C5—C4—N1	119.3 (2)	C12—C11—H11	119.2
C6—C5—C4	119.56 (19)	N4—C12—C11	122.9 (2)
C6—C5—H5	120.2	N4—C12—H12	118.6
C4—C5—H5	120.2	C11—C12—H12	118.6

### Hydrogen-bond geometry (Å, °)

**Table:**

*D*—H···*A*	*D*—H	H···*A*	*D*···*A*	*D*—H···*A*
N2—H2N···N5^i^	0.91	2.30	3.185 (2)	164
C11—H11···O1^ii^	0.93	2.60	3.300 (3)	133

Symmetry codes: (i) *x*+1/2, −*y*+1/2, *z*+1/2; (ii) *x*−1, *y*−1, *z*.
